# Estimating the avoidable burden of certain modifiable risk factors in osteoporotic hip fracture using Generalized Impact Fraction (GIF) model in Iran

**DOI:** 10.1186/2251-6581-12-10

**Published:** 2013-01-30

**Authors:** Banafsheh Shahnazari, Abbasali Keshtkar, Akbar Soltani, Aria Aghamaleki, Asieh Mansour, Bahar Matin, Sharareh Saghafi, Mahboubeh Dini, Patricia Khashayar, Bagher Larijani

**Affiliations:** 1 Osteoporosis Research Center (ORC), Endocrinology and Metabolism Clinical Sciences Research Institute (ECSI), Tehran University of Medical Sciences, Tehran, Iran; 2 Endocrinology and Metabolism Research Center (EMRC), Endocrinology and Metabolism Clinical Sciences Research Institute (ECSI), Tehran University of Medical Sciences, Tehran, Iran; 3 Golestan Research Center of Psychiatry, Golestan University of Medical Sciences, Gorgan, Iran; 4 Musculoskeletal Disease Office, Non-communicable Disease Committee, Iranian Ministry of Health and Medical Education, Tehran, Iran; 5 EMRC, Shariati Hospital, Kargar St, Tehran, 1411413137 Iran

**Keywords:** Osteoporotic fracture, Fragility hip fracture, Calcium, Vitamin D, BMI, Physical activity, Smoke, Prevalence, Incidence

## Abstract

**Backgrounds:**

The number of hip fractures, the most common complication of osteoporosis, has increased rapidly over the past decades. The goal of this study is to estimate the avoidable burden of certain modifiable risk factor of the condition using the Generalized Impact Fraction (GIF) model, which has been suggested and used by epidemiologists to overcome the drawbacks associated with the use of Attributable Fraction index. In addition to preventing a risk factor or the avoidable fraction of burden, this index can also calculate the change in the burden, when a risk factor is altered.

**Methods:**

International databases were searched through PubMed, CINAHLD, Embase using OVID and Google scholar. National resources were searched through IranDoc, IranMedex, SID and Journal sites. Other resources include abstract books and articles sent to the IOF congress. The following search strategy was used: (“Osteoporotic fracture” OR “Fragility Hip fracture” OR “Calcium” OR “vitamin D” OR “BMI” OR “lean body weight” OR “Physical activity” OR “exercise” OR “Smoke”) AND (“prevalence” OR “incidence” OR “relative risk”) and limited to “humans.”

**Results:**

With regards to different scenarios already explained in modifying the studied risk factors, the greatest impact in reducing the prevalence of risk factors on osteoporotic hip fractures, was seen in low serum vitamin D levels, low physical activity and low intake of calcium and vitamin D, respectively. According to the fact that interventions for low serum vitamin D and low intake of calcium and vitamin D, are related to each other, it can be concluded that implementing interventions to change these two risk factors, in the easy, moderate and difficult scenarios, would result in approximately a 5%, 11% and 17% decrease in the burden of osteoporotic hip fractures, respectively. The addition of interventions addressing low physical activity in the easy, moderate and difficult scenarios, an 8%, 21% and 35% reduction in the burden of osteoporotic hip fractures would be reported, respectively.

**Conclusion:**

Improving serum vitamin D levels, recommending the consumption of calcium and vitamin D supplementations and advocating physical activity are the most effective interventions to reduce the risk of osteoporotic hip fractures.

**Electronic supplementary material:**

The online version of this article (doi:10.1186/2251-6581-12-10) contains supplementary material, which is available to authorized users.

## Introduction

Osteoporosis is the most common metabolic disease of the bone[1, 2]. It is practically known as the silent disease but the main consequence is fragility fractures, thus becomes a global challenge. In year 2000, 9000000 osteoporotic fractures occurred throughout the world; 6.1 * 10^6^ in the hip, 1.7 *10^6^ in the forearm, 1.4*10^6^ in the vertebral column. Hip and vertebral fractures are responsible for increased mortality and morbidity in the elderly[3–5].

Osteoporosis is contributed to more than 300000 hip fractures in America annually, a major portion of these patients require hospitalization and surgical interventions[6, 7]. In the European countries, every thirty minutes, one person experiences osteoporotic fractures[8, 9]. The prevalence of osteoporosis is increasing considerably. From every two Asian Caucasian woman, one develops osteoporotic fracture after menopause. Overall, osteoporotic fractures occur in at least 1 in every 8 people of other races[10–13]. In 1990, about 30% of all hip fractures due to osteoporosis occurred in Asia, and the rate is estimated to reach a higher value (50%) by the year 2050.

There are limited statistical data considering hip fractures due to osteoporosis in Iran. In a study conducted by the Ministry of Health and Medical Education in 2008, the prevalence of osteoporotic fractures in Iran, was less, when compared to other Asian and European countries[14]. Iranian studies have indicated that the prevalence of osteoporosis and osteopenia, in at least one site, is about 22.2% (59.9% in women and 11% in men) in those aged 50 and over. As for those younger than 50, 33% of women and 31.6% of men have reduced bone density. Despite the high prevalence of osteoporosis in Iran, the burden of the disease is not calculated[15, 16]. There is a significant difference in peak bone density and the prevalence of osteoporosis in different countries; One study in Iran showed that the peak bone mass of females is higher than the Japanese, the Canadian, the Hong Kong and the Lebanese females and lower than the Americans[17].

In 2001, the comprehensive study of the prevention, diagnosis and treatment of osteoporosis (IMOS) showed that the BMD values in the Iranians is lower than the Americans and higher than the Japanese[18]. The incidence of hip fracture is also different, it’s lower in Asia and Latin America and seems to be lower in rural areas[17].

Osteoporosis imposes a heavy economic burden on the healthcare system. The annual cost of osteoporotic fracture was 17 billion dollars in England, more than 14 billion dollars in America and about 30 billion dollars in Europe. A study conducted in 2004 indicated that in Iran with a population of more than 70 million, the DALY (disability adjusted life years) due to osteoporosis is 36761 years. It also indicated that the mortality rate among the elderly with hip fracture is 20%. The rate was reported to be highest in the first 6 months after hip fracture and the thereafter reduced with time[17]. The estimated burden of hip fracture in Iran is 0.85% of the world and 2.4% of the Middle East. Risk factors associated with osteoporotic fractures are advanced age, history of previous fracture, fall, treatment with glucocorticoids, family history of hip fracture and current cigarette smoking[5, 19].

From the epidemiological point of view, Attributable Fraction (AF) is an index that has traditionally been used to define the impact fraction of different risk factors associated with a disease or health outcome. The main pitfall of this indicator is that the summation of these Attributable fractions is more than 100%. The GIF (Generalized Impact Fraction also called the generalized attributable fraction) index has been suggested and used by epidemiologists, to overcome this concern. In addition to preventing a risk factor or the avoidable fraction of burden, this index can also calculate the change in the burden, when a risk factor is altered. While AF simply studies complete elimination of a risk factor, the GIF estimates the proportional reduction in disease incidence given a graded reduction in the prevalence of a risk factor[20]. In other words, the index is a measure that generalizes the population attributable fraction (attributable risk) and is defined as the fractional reduction of a disease resulting from changing the current distribution of a risk factor to some modified distribution. We show that the point and variance estimator derived for fixed shift functions can be extended to situations where the shift is a probabilistic function of the actual exposure value. Therefore, for common risk factors and diseases, the impact of a hypothetical reduction in the exposure may reveal an important effect on disease incidence even when risk factor-disease associations are relatively weak[21].

Low consumption of calcium and vitamin D supplements, low serum levels of vitamin D, smoking, physical inactivity and low BMI are five modifiable risk factors of osteoporotic hip fracture which are reported to have a high prevalence in Iran. The goal of the present study is to estimate the avoidable burden of a graded reduction in the prevalence of certain risk factors using the GIF model.

## Materials and methods

This study is composed of two phases:

Narrative literature review was performed as the first phase to gather required information for developing the GIF model. In order to collect information on the prevalence of risk factors, and the correlation between these risk factors and osteoporotic hip fracture.

International databases (PubMed, CINAHLD, Embase using OVID and Google scholar) and National resources (Irandoc, IranMedex, SID and Journal sites) were searched. Other resources including abstract books and articles sent to the IOF congress were also studied. The following search strategy was used for literature search: (“Osteoporotic fracture” OR “Fragility Hip fracture” OR “Calcium” OR “vitamin D” OR “BMI” OR “lean body weight” OR “Physical activity” OR “exercise” OR “Smoking”) AND (“prevalence” OR “incidence” OR “relative risk”) and limited to “humans.”

In this regard, the articles studying at least one of the mentioned five risk factors and their relation with osteoporotic hip fracture (presented as relative risk, odds ratio, risk ratio, rate ratio and hazard ratio) were selected. As for each risk factor, a single article was selected based on the following prioritization criteria:

1. The study conducted in the Iranian community was placed at the top of the agenda in each group. In the absence of such a study, the articles reporting the required information from other Middle Eastern nations, the Caucasians, the White Americans and other countries were included.
2. Systematic review or Meta-Analysis took precedence over other types of studies. In the absence of such articles, Clinical trials, Cohort, Case–control, and Cross-sectional studies were included correspondingly.

It should be noted that in case several articles met our inclusion criteria in each group, the most recent study was included.

Those articles that evaluated osteoporotic fractures in areas other than hip and those regarding non-osteoporotic fractures were excluded.

Considering the dissimilarity in the epidemiological indicators of osteoporosis and osteoporotic fractures in different communities, different genders and different age groups (with priority to the elderly), required data for each risk factor was collected and extracted with respect to this principle:

Gender-specific indicators: All the required data including the prevalence and relative risk of each risk factor and the occurrence of osteoporotic hip fractures was analyzed in men and women separately.

Age-specific indicators: All the data used in this study was specific for different age groups: 1) 50 years and older, osteoporosis rate increases in this age group, particularly the postmenopausal women (IMOS study). 2) 65 years and older, in the absence of studies regarding the first group, this age group was used. 3) 60 years and older, in the absence of studies regarding the previous age groups, this age group was used.

Age standardized indicators: If any of the indicators were presented as separated age groups such as five- or ten-year age groups, these indicators should be standardized for one of the above age groups. To standardize each indicator, direct standardization method with respect to demographic weights according to the census performed by Statistical Center of Iran in 1385 (2006) was used.

Table1,2 outlines the definition, frequency and relative risk of the five studied risk factors in men and women.

**Table 1:** **The relative risk of the 5 risk factors of osteoporotic hip fractures for men and women, the definition and level of each factor has been indicated**

Risk factor	Definition categorization	Age group	(CI) Relative risk for women [reference]	(CI) Relative risk for men [reference]
**Low Ca and Vit D intake**	Less than 1000_mg_ Ca and less than 800_u_ vit D	≥50	(1.03-1.25)	(1.03-1.25)
			1.14[22]	1.14[22]
**Low serum Vit D**	Serum level less than 62,5_nmol/lit_	≥65	(1.12-2.17)	(1.12-2.17)
			1.56[23]	1.56[23]
**Smoking**	Current smokers	≥55	(1.12-1.65)	(1.04-2.43)
			1.36[24]	1.59[24]
**Low physical activity or inactivity**	No activity or activity less than 1 hour of walking per week	≥50	(1.06-2.70)	(1.06-2.70)
			1.70[25]	1.70[25]
**Low BMI**	BMI less than 20 in comparison to normal BMI[26–31]	≥50	(1.23-3.28)	(1.23-3.28)
			1.79[32]	1.79[32]

**Table 2:** **Frequency of certain risk factors based on gender**

Risk factor	Definition	Age group	Prevalence for women [reference]	Prevalence for men [reference]
**Low Ca and Vit D intake**	Less than 1000_mg_ Ca and less than 800_u_ vit D	≥50	84.1%[27]	93.6%[27]
**Low serum Vit D**	Serum level less than 62,5_nmol/lit_ or 25_ng/dl_	≥65	41.2%[28]	52.8%[28]
**Smoking**	Current smokers	≥55	2.6%[29]	17.5%[29]
**Low physical activity or inactivity**	No activity or activity less than 1 hour of walking per week	≥50	67.1%[31]	65.4%[31]
**Low BMI**	BMI less than 20	≥50	7.7%[30]	13.3%[30]

The second phase of the study included the statistical analysis of the collected data using the Generalized Impact Fraction (GIF) model.

GIF=∑i=1nPiRRi−∑i=1nPi'RRi∑i=1nPiRRi

GIF = generalized impact fraction

Pi = proportion of the population in exposure category i (fact)

Pi” = proportion of the population in exposure category after an intervention or other change (counter fact)

RR: relative risk

In this phase, the estimated reduction in hip fracture rate through modifying each risk factor was calculated in both genders in three categories: easy, moderate and difficult. Easy scenario indicates the lowest amount of reduction in the prevalence of the risk factor from fact to counter-fact. The moderate scenario indicates medium amount of change, and the difficult scenario belongs to those with most changes. The counter-fact amounts for each risk factor classified in the scenarios mentioned above, and the reduced burden of osteoporotic fracture related to this reduction is summarized in Tables3,4 and5.

**Table 3:** **Decreased burden of the disease by changing the prevalence (Easy scenario)**

Risk factor	Age group	Prevalence		Prediction		Burden reduction	
		en Women	Men	Women	Men	Women	Men
**Low Ca and Vit D intake**	≥50	84.1%	93.6%	70%	80%	1.8%	1.7%
**Low serum Vit D**	≥65	42.1%	52.8%	35%	45%	3.2%	3.4%
**Smoking**	≥55	2.6%	17.5%	1%	12.5%	0.6%	2.7%
**Low physical Activity or inactivity**	≥50	67.1%	65.4%	60%	60%	3.4%	2.6%
**Low BMI**	≥50	7.7%	13.3%	5%	10%	2.0%	2.4%

**Table 4:** **Decreased burden of the disease by changing the prevalence (Moderate scenario)**

Risk factor	Age group	Prevalence		Prediction		Burden reduction	
		en Women	Men	Women	Men	Women	Men
**Low Ca and Vit D intake**	≥50	84.1%	93.6%	55%	65%	3.7%	3.5%
**Low serum Vit D**	≥65	42.1%	52.8%	25%	35%	7.8%	7.7%
**Smoking**	≥55	2.6%	17.5%	0.5%	1%	0.8%	2.5%
**Low physical Activity or inactivity**	≥50	67.1%	65.4%	45%	45%	10.5%	9.8%
**Low BMI**	≥50	7.7%	13.3%	2.5%	7%	3.9%	4.5%

**Table 5:** **Decreased burden of the disease by changing the prevalence (Difficult scenario)**

Risk factor	Age group	Prevalence		Prediction		Burden reduction	
		en Women	Men	Women	Men	Women	Men
**Low Ca and Vit D intake**	≥50	84.1%	93.6%	40%	50%	5.5%	5.4%
**Low serum Vit D**	≥65	42.1%	52.8%	15%	25%	12.3%	12.0%
**Smoking**	≥55	2.6%	17.5%	0.2%	5%	0.9%	6.7%
**Low physical Activity or inactivity**	≥50	67.1%	65.4%	30%	30%	17.7%	17%
**Low BMI**	≥50	7.7%	13.3%	1.0%	5.0%	5.0%	5.9%

## Results

### Literature review

The relative risk of the five studied risk factors (low intake of calcium and vitamin D supplements, low serum levels of vitamin D, smoking, physical inactivity and low BMI) in osteoporotic fractures was assessed.

- For low intake of vitamin D and calcium (alone or together), a meta-analysis and systematic review was found and used as the source for analysis [20]. This article reviewed 17 clinical trials that had evaluated the correlation between low calcium and vitamin D intake and osteoporotic hip fractures. The studied population was aged 50 years and over. Based on the findings of this meta-analysis, appropriate intake of calcium (1000 mg daily) and vitamin D (800 IU daily) lowered the risk of osteoporotic hip fractures by 12%.
- As for vitamin D deficiency, a meta-analysis and systematic review was found [21]. In this systematic review, seven clinical trials (studying the effects of vitamin D supplements on hip fracture), 17 case control studies and four cohort studies were assessed. Relative risk was considered equal in both sexes.
- As for Smoking, a meta-analysis and systematic review studying women was found. This article surveyed 29 cross sectional and 19 analytical (case control and cohort studies). Those aged 55 and older were evaluated. Based on this meta-analysis, smokers had a 36% higher risk of osteoporotic hip fractures. As for men, no systematic review was found and therefore the data of a cohort study were applied instead [24]. Based on this study, the risk of osteoporotic hip fractures was 60% higher in male smokers.
- As for low physical activity and its correlation with osteoporotic hip fractures, no meta-analysis or systematic review was found for women; therefore a prospective cohort, nurse health study, was used [25]. Based on this study, 4 hours or more of exercising each week (walking with moderate speed) was associated with a 40% reduction in osteoporotic hip fracture. For men, no interventional or observational study was found. Thus the impact of low activity or inactivity in men was considered similar to that found in women.
- Evaluating the effects of BMI, a meta-analysis that had analyzed 60000 people in 12 prospective cohort studies was found [32]. Based on this article, each unit increase in BMI values reduces the risk of osteoporotic hip fractures by 7%. Also in those with BMI values lower than 20, the risk of osteoporotic pelvic fracture was 80% higher than that of those with BMI values in the range of 25–30.

Thereafter, a literature review was performed to estimate the prevalence of certain risk factors in the Iranian community. Results are as follows:

- Low intake of calcium and Vitamin D supplements: an article, that surveyed the compliance with supplements in adults referred to the health centers in west part of Tehran [27], was the only available article in this regard. Based on this article, 84% of the women and 94% of the men had inappropriate intake.
- Vitamin D deficiency: Difference in the prevalence vitamin D deficiency has been reported in various part of the country. The results of the IMOS study in five Iranian cities (Tehran, Tabriz, Mashhad, Booshehr and Shiraz) revealed that 42% of women and 52% of men, aged 65 years and over, had moderate to severe Vitamin D deficiency [28].
- Smoking: In order to assess the prevalence of cigarette smoking among middle-aged Iranians, the data of the Tehran Lipid and Glucose study (2001) was applied. The study that surveyed the pattern of smoking among the adult residents of one of the districts of Tehran, reported that 2.6% of women and 17.5% of men aged 55 and over were smokers [29].
- For BMI, the Golestan cohort study reported that 8% of women and 13% of men aged over 50 have BMI values lower than 20 [30].

In the next step, the prevalence of low calcium and vitamin D consumption was calculated (84.1% in women and 93.6% in men aged 50 years and older). By reducing the prevalence in the easy scenario, the burden of the disease would decrease by 1.80%, if the prevalence of the condition reached 70% in women. As for the moderate and difficult scenario, the burden would decrease by 3.7% and 5.4%, respectively. The application of similar scenarios in men showed that reducing the prevalence of the condition to 80% would cause a 1.7% reduction in the burden of disease in the easy scenario. In the moderate scenario, reducing the prevalence to 65% would reduce the burden by 3.5%. In the difficult scenario, the burden was reduced by 5.4% when the prevalence was reduced to 94.6%. The results are similar in men and women, mainly due to the similarity between the prevalence and RR of the condition.

The prevalence of low serum vitamin D was 42.1% in women and 52.8% in men aged 65 and over. By applying the easy scenario, the prevalence of the disease was reduced to 35% and thereafter the burden of fracture in women was calculated to be as low as 3.2%. As for the moderate and difficult scenario, reducing the prevalence to 25% was associated with a 7.8% and 12.3% decline in the burden. The same procedure was applied to men and showed that reducing the prevalence to 45% in the easy scenario would reduce the burden by 3.4%. As for the moderate and difficult scenario, lowering the prevalence to 25%, would result in a 7.7% and 12% decline in the burden, correspondingly. This indicates that the more reduction in the prevalence of individuals with low serum levels of vitamin D, the higher would be the decline in the burden of fracture. The similarity of the data in men and women seems to be secondary to the resemblance of the relative risk and prevalence in both genders.

The prevalence of smoking was 2.6% in women and 17.5% in men. Reducing the prevalence to 1% in women was associated with a 0.6% decline in the estimated burden in the easy scenario, 0.8% in the moderate scenario, and 0.2% in the difficult scenario. As for men, reducing the prevalence to 12.5% caused a 2.7% reduction in burden in the easy scenario, 4.5% in the moderate scenario, and 6.7% in the difficult scenario. The differences between men and women resulted from the higher prevalence and RR of the condition in men.

About 67.1% of women and 65.4% of men are engaged in low physical activity. Based on the easy scenario, reducing the prevalence to 60% would cause a 3.4% and 2.6% decline in the burden of fracture in both women and men aged 50 years and older, respectively. In the moderate scenario, the burden would reduce to 89.5% in women and 90.2% in men after reducing the prevalence to 45%. As for the difficult scenario, however, the prevalence of 30% resulted in an estimated burden of 82.3% and 83% in women and men, respectively. This clarifies that getting engaged in more physical activity would reduce the burden of osteoporotic fracture in both genders.

Based on previous studies, 13.3% of women and men aged 50 and over have low BMI values (lower than 20 kg/m^2^). Reducing the prevalence to 10% in the easy scenario reduced the burden of fracture by 2.4%. The prevalence of 6% in the moderate scenario reduced the burden by 4.5%. The reduction of the prevalence to 5% in the difficult scenario caused a 5.9% reduction in the burden. The results are similar in men and women, mainly due to the similarity of the prevalence and RR rates.

**Figure 1:**
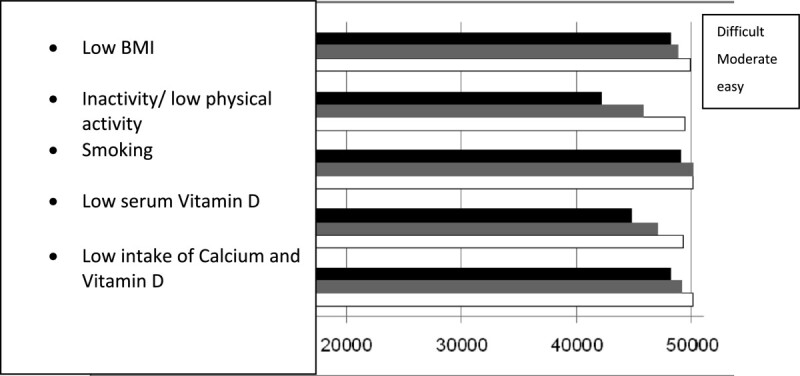
**Reduction in the burden of fracture after reducing the prevalence of the risk factors in the easy, moderate, and difficult scenarios.**

**Figure 2:**
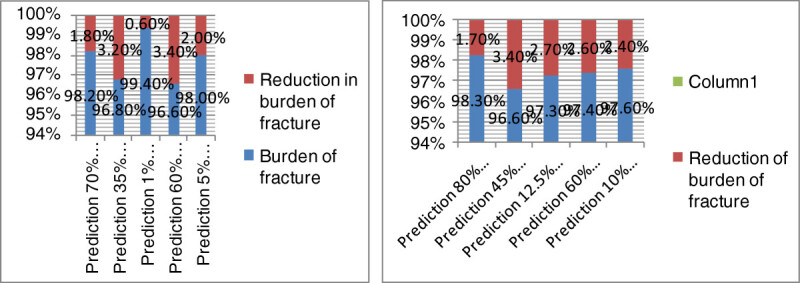
**Reduction in the burden of fracture after reducing the prevalence of the risk factors in the easy scenario in women (a) and men (b) aged 50 years and older.**

Figures1,2, and3 shows the reduction of burden after the application of the easy, moderate, and difficult scenarios (for the five mentioned risk factors) in both women and men aged 50 years and older. In all these situations, the highest reduction in the burden was reported after reducing the prevalence of being engaged in low physical activity, having low serum vitamin D levels, low calcium and vitamin D intake and smoking in women, respectively. Figure4 illustrates the reduction in the burden of fracture for the 3rd prediction of the decrease in risk factors in women aged 50 years and older.

**Figure 3:**
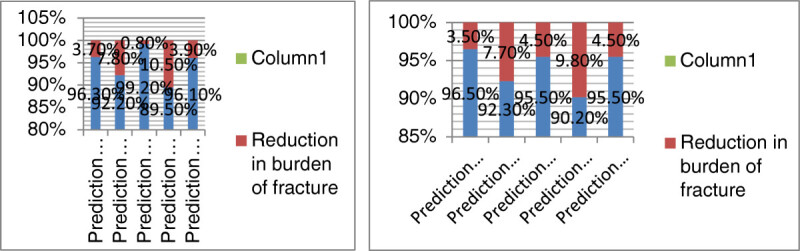
**Reduction in the burden of fracture after reducing the prevalence of the risk factors in the moderate scenario in women (a) and men (b) aged 50 years and older.**

**Figure 4:**
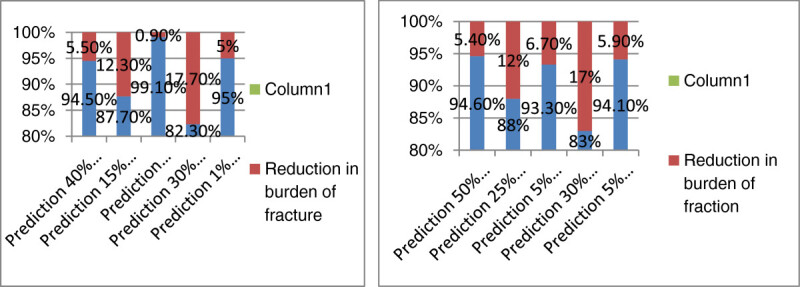
**Reduction in the burden of fracture, for the 3rd prediction of the decrease in risk factors in women aged 50 years and older.**

## Discussion

In order to demonstrate the effects of each risk factor on osteoporotic hip fracture incidence, four main causative models are defined, each has its’ own strengths and weaknesses. These models are listed below:

1. Graphical model 2) Sufficient component cause model 3) Structural equations model 4) Counterfactual model (GIF)

The first two models are qualitative models, but as for evidence based prioritization, quantitative models are needed. While the power and the prevalence of the risk factor are critical, the third model evaluates the power of a risk factor. In the fourth model, the effects of each risk factor on the incidence of the disease are measured.

Murray and Lopez explained four ways to determine the counterfactual status: 1) Reduce the prevalence of a risk factor to the lowest theoretically possible level. (i.e., reducing the prevalence of smoking to zero in a community) 2) Reduce the prevalence of a risk factor to the lowest logically possible level (i.e., the level should be realistic) 3). Reduce the prevalence of a risk factor to the lowest practically possible level (i.e., there are actually some communities in the world, with this level of the risk factor). 4) Reduce the prevalence of the risk factor to the lowest degree that is cost benefit.

It’s obvious that if the goal of an intervention is to reduce the prevalence of a risk factor, considering these four rules one by one, there would be less difference between the current status and the desired level. It seems that using the counterfactual model, the impact of each risk factor can be analyzed. This model identifies the research needs in cases when the available information is not satisfactory, in addition to clarifying the needed data in order to set the points of intervention. With appropriate strategy selection, this kind of analysis can lead to detailed planning for health interventions. Designing and implementing the pilot study leads to the identification of the operational requirements, barriers and related solutions in different field.

This study is the first research that has used the GIF model for evaluating the effects of modifying certain risk factors in the incidence of osteoporotic hip fractures, and no studies have been published about this issue. However, three study was found about GIF model: 1) The Potentially Modifiable Burden of Incident Heart Failure Due to Obesity in USA[33], 2) HIV risk factors in Iran; systematic review, meta-analysis and GIF approaches[34], 3) causal the composition of risk factors in osteoporosis burden[35]. In third research, studied risk factors were smoking, low calcium intake, low physical activity, glucocorticoid consumption and low sun exposure. This study concluded that Interventions for improving physical activity, sun exposure are the best approach for reduction of osteoporosis burden in Iran. And also, in our study physical inactivity has the most effect on osteoporotic hip fracture burden. It revealed that interventions to improve in the serum vitamin D level, the consumption of calcium and vitamin D supplements and physical activity are the most effective interventions to reduce the risk of osteoporotic hip fracture.

For evaluating the impact of smoking, the effects of interventions in reducing the prevalence of smoking is trivial especially in women. These limited effects can be attributed to the low prevalence of smoking in the middle-aged men and especially middle-aged and elderly women in comparison with other risk factors. Intervention for the reduction of smoking require to behavioral modification is hard due to poor compliance in this age group.

In order to evaluate the effects of low BMI (less than 20), since there is no clear and distinctive intervention and any intervention that persuades increasing the BMI in this age group, regardless of the effectiveness, can potentially lead to chronic and non-communicable diseases such as diabetes mellitus, cardiovascular diseases and etc. Therefore this risk factor cannot be manipulated easily in the community.

Iran is a country with high prevalence of moderate to severe vitamin D deficiency in both genders. Vitamin D deficiency is highest among people who are elderly. Insufficient vitamin D intake, air pollution[17] skin complexion, poor sun exposure, vegetarian food habits and lack of vitamin D fortification program can explain the high prevalence of vitamin D deficiency in Iran[28]. Pooled analyses in 2012 suggest that high-dose vitamin D supplementation (≥800 IU daily) was somewhat favorable in the prevention of hip fracture and any non-vertebral fracture in persons 65 years of age or older. Furthermore, the data support a 25-hydroxyvitamin D level above 60 nmol/L for prevention of the fractures[36].

Therefore adequate calcium and vitamin D through diet or supplements, taken together, are essential adjuncts to prevent osteoporotic hip fracture. In order to achieve this goal, increase public awareness, educational programs and persuade multidisciplinary cooperation of various governmental and nongovernmental agencies are recommended. General population and the government should be informed that prevention of osteoporotic hip fractures, costs less than their burden to the society. Encouraging people to regular physical activities and allocating public places to exercise as a national plan can be helpful and needs cooperation of various organizations.

### Limitation

The main limitations of this study and the GIF in general is the fact that an adjusted effect measure cannot be used in the GIF formula (while vitamin D deficiency directly affects fracture risk, BMI influences the outcome indirectly and through affecting a chain of intermediate variables); rather, one must stratify by important confounders and use the crude effect measure for each stratum. In addition, it is difficult to determine feasible goals and interventions for the whole population; for instance while strategies to improve vitamin D levels can be easily implemented in a country, it is difficult to determine feasible goals for weight changes. Another limitation of our study is lack of enough information regarding the prevalence and the relative risk of the certain risk factors of osteoporotic hip fracture. In order to get more information about the prevalence of osteoporosis and the burden of the disease, more accurate planning is mandatory.

## Conclusion

According to the fact that interventions for low serum vitamin D and low intake of Calcium and Vitamin D, are related to each other, it can be concluded that with designing and implementing interventions for changing these two risk factors, in the easy, moderate and difficult scenario, results in approximately 5%, 11% and 17% decrease in the burden of osteoporotic hip fractures, respectively. If interventions for changing low physical activity is added to these interventions, in the easy, moderate and difficult scenarios, 8%, 21% and 35% reduction in the burden of osteoporotic hip fractures is seen, respectively.

## Author’s contribution

All authors read and approved the final manuscript.

## Authors’ original submitted files for images

Below are the links to the authors’ original submitted files for images. Authors’ original file for figure 1 Authors’ original file for figure 2 Authors’ original file for figure 3 Authors’ original file for figure 4
