# Supplementary material for: Estimating the avoidable burden of certain modifiable risk factors in osteoporotic hip fracture using Generalized Impact Fraction (GIF) model in Iran
Source: J Diabetes Metab Disord. 2013 Jan 30;12:10. doi: 10.1186/2251-6581-12-10 (PMC3598997; doi:10.1186/2251-6581-12-10)

- Low BMI
- Inactivity/ low physical activity
- Smoking
- Low serum Vitamin D
- Low intake of Calcium and Vitamin D

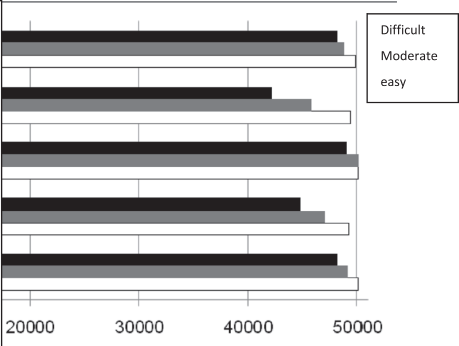

Supplement: Supplementary file 1 — Authors’ original file for figure 1 [file 40200_2012_38_MOESM1_ESM.pdf]
