# Supplementary figures and images for: Estimating the avoidable burden of certain modifiable risk factors in osteoporotic hip fracture using Generalized Impact Fraction (GIF) model in Iran
Source: J Diabetes Metab Disord. 2013 Jan 30;12:10. doi: 10.1186/2251-6581-12-10 (PMC3598997; doi:10.1186/2251-6581-12-10)

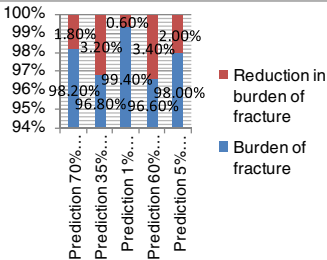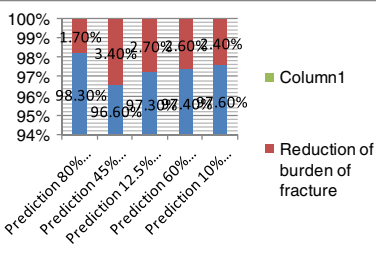

Supplement: Supplementary file 2 — Authors’ original file for figure 2 [file 40200_2012_38_MOESM2_ESM.pdf]

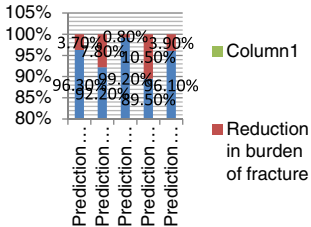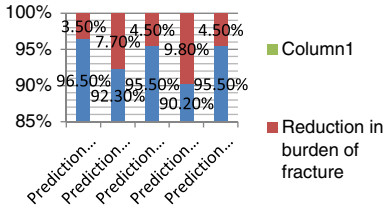

Supplement: Supplementary file 3 — Authors’ original file for figure 3 [file 40200_2012_38_MOESM3_ESM.pdf]

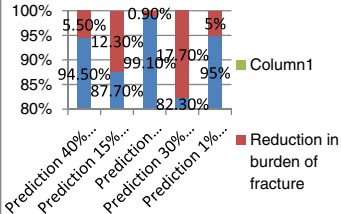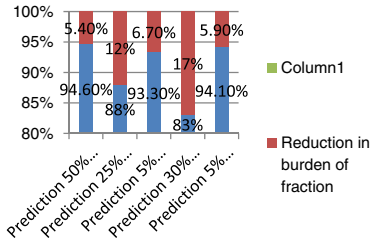

Supplement: Supplementary file 4 — Authors’ original file for figure 4 [file 40200_2012_38_MOESM4_ESM.pdf]
